# Investigation of Community Integration in Adults with Congenital Heart Disease Within the Scope of International Classification of Functioning, Disability, and Health

**DOI:** 10.1007/s00246-025-03850-4

**Published:** 2025-04-05

**Authors:** Tugba Siyah, Ceyhun Topcuoglu, Naciye Vardar Yagli, Ebru Calik Kutukcu, Hayrettin Hakan Aykan, Ilker Ertugrul, Tevfik Karagoz, Melda Saglam

**Affiliations:** 1 Faculty of Physiotherapy and Rehabilitation, Hacettepe University, Ankara, Turkey; 2 Department of Pediatric Cardiology, Faculty of Medicine, Hacettepe University, Ankara, Turkey

**Keywords:** Congenital heart disease, Community Integration, ICF, Participation

## Abstract

The number of adults living with congenital heart disease continues to rise, emphasizing the need to understand the challenges they encounter to enhance disease management. This study aimed to evaluate adults with CHD using the International Classification of Functioning (ICF) framework and explore factors associated with community integration. Holistic assessments were conducted encompassing all dimensions of the ICF framework, including Body Structures (echocardiography and electrocardiography findings), Body Functions (6-Minute Walk Test, Muscle Strength, Hand Grip Strength, and Fatigue Severity Scale), Activity-Participation (International Physical Activity Questionnaire, Multidimensional Quality of Life Scale, and Community Integration Questionnaire), and Environmental-Personal factors (Depression, Anxiety, and Stress Scale- 21, and Physical Activity Barriers Scale). Pearson correlation analysis was conducted to examine the relationships between community integration and other parameters, with variables showing significant correlations included in the multiple linear regression analysis. The mean age of 42 CHD participants was 24.61 ± 7.27 years. The Community Integration Questionnaire correlated with the Physical Activity Barriers Scale (*r* = 0.310, *p* = 0.046), Multidimensional Quality of Life Scale (*r* = 0.441, *p* = 0.003), and 6-min walk test (*r* = 0.364, *p* = 0.021). “Access to health personnel,” a sub-dimension of the Multidimensional Quality of Life Scale, and 6-min walk test distance predicted the Community Integration Questionnaire score, explaining 30.7% of its variance (*r* = 0.554; *r*^2^ = 0.307; F = 8.197; *p* = 0.001). The findings suggest that community integration in individuals with CHD is linked to body function, activity-participation, and environmental-personal factors. This study highlights the importance of evaluating patient-reported outcome measures in the domain of community integration, especially considering the shifting demographics of congenital heart disease.

**Supplementary Information:**

The online version contains supplementary material available at 10.1007/s00246-025-03850-4.

## Introduction

Congenital heart disease (CHD) is one of the common birth defects with a prevalence of approximately 1 in 1000 live births worldwide [1]. In the past years, about 15% of patients with CHD reached adulthood. However, due to advancements in medicine and technology, this rate has significantly increased to nearly 90% today [2]. Recent years have seen a dramatic decrease in mortality rates following congenital heart surgeries performed in pediatric heart centers, now falling to between 2 and 3% [3]. This reduction is attributed to improvements in diagnosis, surgical techniques, myocardial protection using cardiopulmonary bypass, advanced postoperative monitoring, and the use of extracorporeal membrane oxygenation [4]. Today, the number of adults living with CHD surpasses the number of children with the condition. This shift poses unique challenges and gaps in healthcare systems, emphasizing the need for specialized care and lifelong follow-up for adults with congenital heart disease (ACHD) [5].

Despite advancements in medicine and technology that allow individuals with CHD to survive into adulthood, they may still face lifelong challenges associated with the disease. As patients with CHD age, they are at an increased risk of developing acquired cardiovascular risk factors common in the general population, such as hypertension, obesity, and diabetes. These factors can lead to metabolic disease, stroke, and coronary artery disease [6]. The impact of CHD on quality of life can be significant, influenced by factors, such as difficulties in treatment and disease management, social and physical limitations, repeated treatments and surgical interventions, future health concerns, and the need for lifelong follow-up. Beyond the individual impact, the disease also imposes a global burden due to direct healthcare costs and potential or actual loss of productivity [7]. As the number of adults living with CHD increases, it becomes imperative to identify the challenges they face and tailor healthcare services to meet their specific needs. One significant challenge is community integration, which refers to an individual’s capacity to function autonomously within their environment, fulfill age-appropriate social roles, actively participate in society, and engage in productive behaviors [8]. Studies on community integration primarily focus on individuals with cerebrovascular accidents, traumatic brain injury, intellectual and developmental disabilities, mental illnesses, and older adults with chronic diseases [9]. Community integration has been reported to enhance overall health and well-being, reduce the likelihood of secondary disabilities, and improve quality of life across all populations and settings [10]. However, there is a clear knowledge gap exists concerning ACHD population.

Community integration is essential for empowering individuals to become productive and valued members of society, fostering independence in life choices, and reducing the burden on societal resources. It aligns with the fundamental principle that all individuals, irrespective of their circumstances, deserve equal opportunities to live, work, socialize, and engage in leisure activities [11]. Notably, community integration is strongly associated with quality of life, particularly among individuals with chronic illnesses or physical limitations [12]. Therefore, assessing community integration is essential to guiding individuals toward suitable and productive alternatives based on their level of social participation [13]. A holistic understanding of health, extending beyond individual and physical aspects to encompass environmental factors and social connections, is crucial. Treatment strategies should therefore integrate individuals’ social support networks to promote this comprehensive approach [9]. A holistic approach to understanding these problems is essential to address the key issues that require the attention of clinicians.

The International Classification of Functioning, Disability and Health (ICF) is an international, standardized classification system that provides a common language and framework for defining health and health-related conditions [14]. It emphasizes that chronic diseases should not be viewed solely as medical problems but also in terms of the functional limitations and disabilities they cause. ICF, which is a holistic assessment system, consists of two parts: “functionality and disability” and “contextual factors.” The functionality and disability section includes body structures, body functions, activity, and participation. The contextual factors includes environmental factors and personal factors [15]. The ICF offers a comprehensive framework for understanding the interactions among body structure, body functions, activity-participation, environmental, and personal factors. This approach allows for information collection from various perspectives, providing a comprehensive assessment of the disease’s impact.

Existing literature lacks studies that evaluate body structures-functions, activity-participation levels, and environmental-personal factors in adults with congenital heart disease using the ICF framework. Individuals with ACHD may experience difficulties in community integration due to physical and psychosocial difficulties caused by CHD. Therefore, the aim of this study was to evaluate adults with CHD within the ICF framework and to investigate the parameters related to community integration.

## Material and Methods

### Study Design

This cross-sectional study was conducted between September 2022 and September 2023 at Hacettepe University, Faculty of Physical Therapy and Rehabilitation, Department of Cardiorespiratory Physiotherapy and Rehabilitation. The study was approved by Hacettepe University, Non-Interventional Clinical Research Ethics Committee with the registration number GO 22/972. Clinical Trial registration number is NCT05774158.

Adults aged 18–65 years with a stable clinical condition and diagnosed by a cardiologist as having simple, moderate, or complex CHD as classified by the current American Heart Association (AHA) guidelines, were eligible for inclusion in the study. Participants with neurologic, orthopedic, or systemic diseases, active infections, malignancies, cognitive impairments that would hinder assessment, non-cardiac surgeries, and recently planned cardiac surgery were excluded. After receiving detailed information about the study, participants provided informed consent.

### Assessments Based on ICF Framework

Demographic and physical characteristics of the patients were recorded. The evaluation methods used within the framework of ICF were as follows:

#### Body Structure

CHD diagnoses were recorded based on echocardiography and electrocardiography findings performed by a specialist physician and classification was made according to the types of CHD. The disease severity of the participants was classified as simple, moderate, and complex according to the current AHA guidelines [16] (Table 1).

**Table 1:** Characteristics of the patients

	Mean ± SD	Min–Max
Age (years)	24.6 ± 7.2	18–47
Height (cm)	170 ± 10.2	151–200
Body mass index (kg/m^2^)	22.8 ± 3.8	17.6–35.8

	*n*	%
Type of Heart Disease		
Simple CHD	7	16.7
Moderate CHD	24	57.1
Complex CHD	11	26.2
Surgical History		
Yes	39	92.9
None	3	7.1
Educational Status		
Primary education	2	4.8
High school	19	45.2
University	21	50

*CHD* congenital heart disease, *cm* centimeter, *kg* kilogram, *m*^2^ square meter, *SD* standard deviation

#### Body Functions

The 6-min walk test (6MWT) was used to evaluate the functional capacity and exercise response of participants. The 6MWT is widely recognized, easy to perform, and safe submaximal exercise test for individuals with CHD [17]. For the 6MWT, a 30-m walking area was marked and participants were instructed to walk this distance for 6 min at their maximum comfortable pace. Fatigue levels were assessed using the modified Borg scale, while blood pressure, heart rate, and oxygen saturation (Nonin Palmsat 2550 A, Nonin Medical Inc., Plymouth, MN, USA) were recorded both before and after the test. The reference values for the 6MWT range between 410 and 875 m for males and 383 and 800 m for females [18].

Knee extensor and shoulder abductor muscle strength were evaluated using a digital dynamometer (Lafayette Instrument Inc., Lafayette, IN, USA). Muscle strength measurements were performed three repetitions from both right and left side extremities of the participant and the mean values were recorded [19]. Maximum isometric hand grip strength was evaluated using an electronic hand dynamometer (Jamar, Sammons Preston, Rolyon, Bolingbrook, IL, USA). Three measurements were performed separately for the right and left hand and the average was calculated. A rest period of 20 s was allowed between measurements. According to reference values, knee extension muscle strength ranges from 39.4 to 58.6 to kg in adult males and from 27.8 to 47.6 kg in adult females. Shoulder abductor muscle strength ranges from 20.7 to 26.3 kg in adult males and from 11.4 to 15.6 kg in adult females. Handgrip strength ranges from 41 to 55 kg in adult males and from 23 to 32 kg in adult females. Expected values for peripheral muscle strength and hand grip strength were calculated based on age, sex, and weight using reference equations [20, 21].

The Fatigue Severity Scale was used to assess the impact of fatigue levels on functioning. Participants rate each question on a scale of 1–7, reflecting their experiences over the past week. The total score is calculated by dividing the sum of the scores by the number of questions. A score of 4 or higher generally indicates severe fatigue [22].

#### Activity and Participation

The International Physical Activity Questionnaire (IPAQ) was used to assess the physical activity level of the participants. The questionnaire focuses on physical activity levels over the past 7 days. Based on the total physical activity score, participants were categorized as inactive, minimally active, or sufficiently active. Additionally, sitting time was recorded [23].

The Multidimensional Quality of Life Scale was used to assess the quality of life of participants. This 35-item scale measures six sub-dimensions: mental health, physical health, social functioning, access to health personnel, interpersonal communication, and financial status. Scores range from 7 to 245 points, with higher scores indicating better quality of life [24].

The Community Integration Questionnaire was used to assess participation in the study. The questionnaire consists of home participation, social participation, and participation in productive activities. Home participation scores range from 0 to 10, social participation scores from 0 to 12, and productive activities scores from 0 to 7. Scoring focuses on both the frequency of performing activities or social roles and whether they are performed alone or with family members. The highest possible score is 29, with a higher score indicating increased community integration [25].

#### Environmental and Personal Factors

The 21-item short form of the Depression, Stress, and Anxiety scale was used to measure depression, anxiety, and stress levels in participants. The scale includes seven questions for each dimension (depression, stress, and anxiety) using a 4-point Likert scale. Scores of 0–9 indicate normal depression levels, 0–7 for anxiety, and 0–14 for stress. Higher scores suggest increasing levels of depression, stress, and anxiety, ranging from normal to quite severe [26].

The Physical Activity Barriers Scale assessed the factors limiting participants’ physical activity. This 22-item scale measures personal factors, social environment, and physical environment that hinder physical activity. Scores range from 22 to 100, with higher scores indicating greater barriers to physical activity [27].

ICF codes associated with the assessment parameters are presented in Supplemental Table 1.

### Statistical Analyses

Data were analyzed using SPSS 22.0 (IBM, Chicago, IL, USA). Descriptive statistics were expressed as mean and standard deviation. Pearson correlation analysis was used to analyze the relationships between community integration and other parameters. Correlation coefficients were interpreted as follows: 0.30–0.40 as low-moderate, 0.40–0.60 as moderate, 0.60–0.70 as good, 0.70–0.75 as strong, and 0.75–1.00 as very strong [28]. Variables significantly correlated with the Community Integration Questionnaire in the Pearson correlation test were included in the multiple linear regression analysis. Stepwise regression analysis was used for variable selection, including all variables with *p* < 0.01 in the univariate regression model. The significance level was accepted as *p* < 0.05.

## Results

A total of 69 individuals were invited to participate in the study, of which 23 declined. Four individuals were excluded due to planned surgeries. Ultimately, 42 adults with ACHD were included in the study. The mean age of the participants was 24.6 ± 7.2 years, and the mean body mass index (BMI) value was 22.8 ± 3.86 kg/m^2^.

### Body Structures

The study population included 45.2% (*n* = 19) with tetralogy of Fallot, 23.8% (*n* = 10) with transposition of the great arteries, and 9.5% (*n* = 4) with ventricular septal defects (VSD). Additionally, there were patients with aortic valve replacement, hypoplastic left heart syndrome, Ebstein’s anomaly, atrial septal defects (ASD), aortic insufficiency, and aortic coarctation. All included patients were clinically stable and under regular follow-up with a cardiologist.

Based on the current AHA guidelines, among the participants in our study, 7 (16.7%) had simple CHD, 24 (57.1%) had moderate CHD, and 11 (26.2%) had complex CHD (Table 1).

### Body Functions

The total distance covered in the 6MWT by participants to evaluate functional capacity was 521.7 ± 90.1 m. According to a reference equation, the 6MWT walking distance of individuals with ACHD is 65.5% of the expected value [29]. The findings of 6MWT are shown in Table 2.

**Table 2:** Body functions

	Mean ± SD	Min–Max
6MWT		
6MWT total distance (m)	521.7 ± 90.1	323–710
Post-exertion heart rate (beats/min)	130.7 ± 22.4	86–169
Post-exertion oxygen saturation (%)	91.3 ± 8.2	70–99
Shortness of breath post-exertion-Modified Borg (0–10 points)	2.7 ± 2.2	0–8
General fatigue post-exertion-Modified Borg (0–10 points)	3.1 ± 2.2	0–8
Muscle Strength		
Right knee extensor (kg)	23.5 ± 7.6	13.5–39
Left knee extensor (kg)	22.6 ± 6.2	13.5–39.2
Right shoulder abductor (kg)	18.5 ± 6.9	9.3–33.9
Left shoulder abductor (kg)	17.9 ± 6.6	9–30.4
Right-hand grip (kgf)	31.9 ± 10.7	17.3–51.3
Left-hand grip (kgf)	30.4 ± 10.1	16.6–52.3
Fatigue Severity Scale (1–7 points)	4 ± 1.9	0–7

*6MWT* 6-min walk test, *kg* kilogram, *kgf* kilogram-force, *m* meter, *min* minute, *SD* standard deviation

The peripheral muscle strength values were 23.5 ± 7.6 kg for right knee extensors, 18.5 ± 6.9 kg for right shoulder abductors, and 31.9 ± 10.7 kg for right handgrip strength. According to the reference equations, the knee extensor strength of individuals with ACHD was 45.2% of the expected value, shoulder abductor strength was 90.9%, and handgrip strength was 54.1%.

Based on the Fatigue Severity Scale score, 52.3% of participants reported severe fatigue (Table 2).

### Activity and Participation

The total score of the Multidimensional Quality of Life Scale was 180.1 ± 36.7.

The weekly total physical activity score of the participants was 1944.5 ± 1915.2 MET-min/week. Of the participants, 33% were inactive, 40.5% had low physical activity level, and 23.8% had sufficient physical activity level. The total sedentary time was approximately 7 h.

The total score of the Community Integration Questionnaire was 15.8 ± 4.2 points (Table 3).

**Table 3:** Activity-participation and personal-environmental factors

Activity and participation		
	Mean ± SD	Min–Max
Multidimensional Quality of Life Scale		
Total Score (7–245 points)	180.1 ± 3676	80–233
Physical Health	75.6 ± 15.5	43–100
Mental Health	20.2 ± 5.2	10–28
Relationship with Health Professionals	30.1 ± 4.6	12–37
Interpersonal Relations	25.9 ± 7.6	9–36
Financial Status	13.8 ± 5.0	0–21
Social Functioning	10.1 ± 3.7	2–14
International Physical Activity Questionnaire (IPAQ)		
Total PA Score (MET-min/week)	1944.5 ± 1915.2	132–7065
Vigorous PA (MET-min/week)	400.2 ± 957.3	0–3600
Moderate PA (MET-min/week)	317.8 ± 645.0	0–2880
Walking activity (MET-min/week)	1373.2 ± 1329.2	99–5544
Sitting time (min)	425 ± 186.2	120–870
Community ıntegration questionnaire		
Total Score (0–29 points)	15.8 ± 4.2	5.5–25.7
Home Integration (0–10 points)	4.5 ± 2.3	1.2–10
Social Integration (0–12 points)	7.5 ± 2.1	3–11
Integration into Productive Activities (0–7 points)	3.7 ± 1.9	0–6

Personal and environmental factors		
	Mean ± SD	Min–Max
Physical Activity Barriers Scale (22–100 points)	51.2 ± 14	22–80
Depression Anxiety Stress Scale		
Depression (0–21 points)	7.6 ± 8	0–42
Anxiety (0–21 points)	7.6 ± 6.3	0–28
Stress (0–21 points)	8.8 ± 7.7	0–42

*MET* metabolic equivalent, *min* minute, *PA* physical activity, *SD* standard deviation

### Environmental and Personal Factors

The total score of the Physical Activity Barriers Scale was 51.2 ± 14 points. The items with the highest scores were as follows: ‘I have health problems that prevent me from being physically active,’ ‘I do not have friends with whom I can do physical activities,’ ‘I do not have free time to exercise or do physical activity because of my job,’ ‘I do not have extra energy to do physical activity after finishing my job,’ and ‘Physical activity is difficult and exhausting.’

The participants in our study were categorized based on their depression levels as follows: 66.7% normal, 14.3% mild, 9.5% moderate, 4.8% severe, and 2.4% extremely severe. Their anxiety levels were 61.9% normal, 2.4% mild, 19% moderate, 9.5% severe, and 4.8% extremely severe. Stress levels were 83.3% normal, 7.1% mild, 4.8% moderate, and 2.4% severe (Table 3).

### The Relationship Between ICF Parameters and Community Integration

A low-moderate negative correlation was found between the total score of the Community Integration Questionnaire (activity-participation dimension of ICF) and the Physical Activity Barriers Scale (personal-environmental factors dimension) (*r* = − 0.310, *p* = 0.046). There was no significant relationship between the Community Integration Questionnaire and depression (*r* = − 0.286, *p* = 0.070), stress (*r* = − 0.173, *p* = 0.280), or anxiety (*r* = − 0.176, *p* = 0.271) levels.

There was a moderate positive relationship between the Community Integration Questionnaire and the total score of the Multidimensional Quality of Life Scale in the activity-participation dimension (*r* = 0.441, *p* = 0.003). There was no significant relationship between the Community Integration Questionnaire and physical activity score (*r* = 0.275, *p* = 0.081).

A low-moderate positive correlation was found between the Community Integration Questionnaire and the total distance covered in the 6MWT (body functions dimension) (*r* = 0.364, *p* = 0.021). There was no significant relationship between the Community Integration Questionnaire and knee extensor muscle strength (*r* = 0.238, *p* = 0.134), shoulder abductor muscle strength (*r* = 0.196, *p* = 0.220), hand grip strength (*r* = 0.137, *p* = 0.389), and Fatigue Severity Scale (*r* = 0.083, *p* = 0.600) (Table 4).

**Table 4:** Factors correlated with community ıntegration questionnaire

	Community ıntegration questionnaire		Community ıntegration questionnaire-social ıntegration	
	*r*	*P*	*r*	*P*
6MWT total distance (m)	.364	.021*	.361	.022*
Knee extensor strength (Right, kg)	.238	.134	.412	.007*
Shoulder abductor strength (Right, kg)	.196	.220	.281	.075
Hand grip strength (Right, kg)	.137	.389	.238	.129
Multidimensional Quality of Life Scale	.441	.003*	.455	.002*
Physical Health	.215	.177	.298	.058
Mental Health	.330	.035*	.553	< 0.001**
Relationship with Health Professionals	.499	.001**	.403	.009*
Interpersonal Relations	.301	.055	.443	.004*
Financial Status	.352	.024*	.311	.047*
Social Functioning	.284	.072	.421	.006*
Fatigue Severity Scale	.083	.600	−.063	.693
IPAQ total score	.275	.081	.452	.003*
Physical Activity Barriers Scale	−.310	.046*	−.469	.002*
Depression	−.286	.070	−.474	.002*
Anxiety	−.173	.280	−.344	.028*
Stress	−.176	.271	−.375	.016*

*6MWT* 6-min walk test, *IPAQ* ınternational physical activity questionnaire, *kg* kilogram, *m* meter; *r* Pearson correlation coefficient

*p < 0.05; **p < 0.001

In addition to the correlations observed with the Community Integration Questionnaire, the sub-dimension of this questionnaire, Social integration, also demonstrated significant correlations with knee extensor strength (*r* = 0.412, *p* = 0.007), interpersonal relations (*r* = 0.443, *p* = 0.004), social functioning (*r* = 0.421, *p* = 0.006), IPAQ (*r* = 0.452, *p* = 0.003), depression (*r* = − 0.474, *p* = 0.002), anxiety (*r* = − 0.344, *p* = 0.028), and stress (*r* = − 0.375, *p* = 0.016) (Table 4).

The variables obtained by stepwise method to obtain the multiple regression model are shown in Table 5. ‘Access to health personnel’ scores (Standardized Beta = 0.432), which is sub-dimension of the Multidimensional Quality of Life Scale and 6MWT total distance (Standardized Beta = 0.252), are significant predictors of the total score of Community Integration Questionnaire. When combined, they explain 30.7% of the variance in the Community Integration Questionnaire total score (*r* = 0.554; *r*^2^ = 0.307; F = 8.197; *p* = 0.001). In addition, ‘mental health,’ which is a sub-dimension of the Multidimensional Quality of Life Scale, is a significant predictor of the ‘social integration’ sub-dimension of the Community Integration Questionnaire. It explains 30.5% of the variance in social integration (*r* = 0.553; *r*^2^ = 0.305; F = 17.150; *p* < 0.001).

**Table 5:** Univariate and multiple regression analysis results to determine the factors affecting the total score of the Community Integration Questionnaire

	Univariate		Multivariate	
	*B*	*p*	*B*	*p*
6MWT total distance (m)	0.017	0.021*	0.014	0.033*
Knee extensor strength (Right, kg)	0.133	0.134		
Shoulder abductor strength (Right, kg)	0.121	0.220		
Hand grip strength (Right, kg)	0.054	0.389		
Multidimensional Quality of Life Scale total score	0.051	0.003*		
Mental Health	0.260	0.035*		
Relationship with Health Professionals	0.448	0.001**	0.420	0.002*
Financial Status	0.289	0.024*		
Social Functioning	0.318	0.072		
Fatigue Severity Scale	0.180	0.600		
International Physical Activity Questionnaire	0.001	0.048*		
Physical Activity Barriers Scale	− 0.094	0.046*		
Depression	− 0.153	0.070		
Anxiety	− 0.116	0.280		
Stress	− 0.097	0.271		
			*R* = 0.554; R^2^ = 0.307; F = 8.197; *p* = 0.001	

*kg* kilogram, *m* meter

*p < 0.05; **p < 0.001

## Discussion

The main findings of our study in individuals with ACHD demonstrate that ICF components are interconnected. Our results reveal that the social integration dimension, a crucial indicator of daily life challenges, is associated with various parameters, including functional capacity, muscle strength, factors preventing physical activity, quality of life, physical activity level, depression, stress, and anxiety. These relationships, particularly within the social integration dimension, are significant as different parameters can contribute to and exacerbate each other’s risks.

### Functional Capacity

Individuals with ACHD face a higher mortality risk than their healthy peers, and impaired functional capacity is a significant contributing factor [30]. Functional capacity is significantly reduced in individuals with ACHD [31]. In alignment with these findings, the 6MWT distances of individuals with ACHD in our study were lower than reference values [32]. A systematic review found that functional capacity in children and adolescents with CHD was lower than in their peers, even after treatment. Impaired chronotropic response was identified as a contributing factor [33]. The literature generally attributes low functional capacity to abnormalities in the cardiovascular system, muscles, chronotropic insufficiency, pulmonary disorders, and cachexia [34, 35]. Researchers have emphasized that functional capacity, which holds prognostic significance, impacts lifestyle, and therapeutic interventions [34]. The results of our study also support this finding. Given the relationship between functional capacity, a crucial marker of body function, and the activity-participation dimension of ICF, we believe that holistic assessment approaches will contribute more effectively to disease management.

### Muscle Strength

Isotonic muscle strength of individuals with ACHD is significantly lower than healthy reference values and this difference increases as the NYHA score increases [36]. It has been reported in the literature that a physically sedentary lifestyle may contribute to decreased muscle function [37]. In our study, we found that the muscle strength of individuals with ACHD was lower than the expected reference values [20]. Additionally, muscle strength was associated with social integration from the activity and participation dimension of the ICF. Few studies have examined the effects of exercise training in individuals with ACHD, but those that have been conducted suggest that a combination of strength training and aerobic exercise can improve cardiac output, exercise capacity, and skeletal muscle function [38]. Further research is needed to investigate the effect of muscle strength training on social integration in individuals with ACHD. Given that muscle function is a predictor of long-term survival [39], individuals with ACHD are at a disadvantage. Therefore, it is crucial to develop rehabilitation approaches that target muscle function improvement.

### Fatigue Severity Scale

Fatigue is a multifaceted phenomenon characterized by a decline in mental and physical working capacity and an intense feeling of exhaustion [40]. There was no relationship between fatigue and community integration, but considering the multidimensional definition of fatigue, it is important to examine the factors related to fatigue in a multidimensional framework. Various factors have been identified as contributing to fatigue in individuals with ACHD, including the degree of physical activity, muscle function, age, and heart failure [41]. Given that individuals with CHD have been living with the disease since birth, they may perceive fatigue, a subjective phenomenon, as a normal condition. Therefore, it is essential to evaluate fatigue using patient-reported outcome measures and consider the associated factors.

### Quality of Life

With increasing life expectancy, serious comorbidities increase the uncertainty of life expectancy. This situation makes quality of life increasingly important. The findings of our study showed that different sub-dimensions of quality of life were associated with community integration. When evaluating quality of life, a multifaceted marker, our study highlights the significance of the ICF model in disease management. Literature on the quality of life of individuals with CHD presents conflicting results. When quality of life is measured holistically in terms of ‘life satisfaction,’ individuals with CHD may report a better quality of life than their healthy peers. However, when measured in terms of ‘physical functioning,’ those with complex CHD defects tend to have worse quality of life than individuals with moderate or mild defects and healthy individuals [42].

A study conducted in adolescents and young adults concluded that the overall quality of life of those with CHD was not lower than that of healthy controls [43]. Another study found that physical function and general health perception were worse in moderate or complex CHD compared to healthy individuals [44]. In a study involving adults with CHD, quality of life was found to be lower than in healthy controls, and this result was not related to demographic or socioeconomic factors. Simply having CHD, even without accompanying complications, can negatively impact an individual’s quality of life. As the severity of the disease increases, the quality of life of individuals with ACHD deteriorates [45]. Despite growing evidence, studies evaluating the quality of life of individuals with CHD still show inconsistencies. Factors such as differences in quality of life measurement tools, research methodology, and the complexity of CHD can hinder definitive results [42]. Since disease-specific factors may worsen over time, early diagnosis and treatment are crucial for disease management and improving quality of life.

### Physical Activity

In our study, 31% of individuals with ACHD were not physically active. Existing studies support the relationship between physical activity level and health-related quality of life in individuals with ACHD [46]. However, there is a lack of literature on community integration. The findings of our study contribute to expanding the existing knowledge in this area, by demonstrating the relationship between physical activity and community integration. Similar to our findings, a study evaluated the physical activity levels of adults with CHD and found that they ranged from normal to severely limited. Additionally, many participants in this study reported being willing to exercise but unsure about its safety or benefits [47]. Traditionally, patients with complex CHD were advised to avoid moderate or intense physical activity due to safety concerns. However, recent research provides evidence that these patients, especially those with highly complex CHD, can benefit from exercise training both physiologically and psychologically [48]. Studies have clearly demonstrated the benefits of physical activity in managing chronic diseases. The European Society of Cardiology also states that regular exercise at recommended levels is feasible and should be encouraged in all patients with CHD [49].

### Barriers to Physical Activity

Children diagnosed with CHD generally show a tendency toward more sedentary behavior and less physical activity participation regardless of the severity of their disease. This situation tends to worsen with age [50]. Various barriers to physical activity participation have been identified, such as social stigmatization and overprotective approach [51]. A qualitative study with adolescents with CHD revealed that environmental factors in the social and school environment significantly impact participation in physical activity. This study highlighted that participation in physical activity can increase social interactions but may also be challenging, leading to feelings of anxiety, frustration, or guilt. The study emphasizes the importance of considering individual factors, family dynamics, geographic environment, and social environment within the ICF model to facilitate the integration of physical activity into daily life [52]. In addition, among the reasons that prevented the participants from being physically active, the items ‘I have health problems that prevent me from being physically active,’ ‘I do not have friends with whom I can do physical activities,’ and ‘I do not have free time to exercise or do physical activity because of my work’ were marked the most. Given the relationship between physical activity barriers and community integration, accurate information about the safety and effectiveness of physical activity in the ACHD population should be provided and physical activity should be encouraged. Additionally, the factors preventing physical activity should be analyzed comprehensively. Studies on physical activity barriers in children and adolescents with CHD should be expanded to include the adult population, as different barriers may be encountered in each age group.

### Depression Stress Anxiety

Individuals with ACHD frequently have to cope with psychologic problems, such as depression, anxiety, or posttraumatic stress disorders [53]. Using a structured clinical interview method with 50 patients with ACHD, a study found that 33% met the criteria for major depressive disorder and 26% met the criteria for anxiety disorder. Using screening tools, this study estimated the prevalence of depression to be between 6 and 22% and the prevalence of anxiety to be between 34 and 42% [54]. Although most participants in our study had normal levels, 31% had depression, 14.3% had stress, and 35.7% had anxiety levels ranging from mild to extremely severe. Depression, stress, and anxiety are related to the social integration dimension of community integration. Unlike individuals with acquired heart disease, the stress of living with heart disease since childhood, undergoing surgeries, paying for medical care, and uncertainty about the need for future interventions may increase with age in CHD [55]. There are very few studies on the diagnosis and treatment of depression and mental disorders in the CHD population. Further studies are needed to analyze these problems in different age and diagnosis groups within the CHD population.

### Community Integration

Developmental impairments that persist in adolescents with CHD are associated with reduced physical and social participation. This relationship may put individuals with CHD at risk of deteriorating physical and mental health [56]. In individuals with CHD aged 24–28 years, the presence of the disease has been reported to significantly affect the reasons for not attending work or school [57]. Our results demonstrate that community integration is associated with mental health factors (depression, stress, anxiety) and physical health factors (muscle strength, functional capacity). Additionally, community integration is linked to physical activity barriers, financial status, interpersonal communication, and access to health personnel.

Moreover, the examination of factors associated with social integration emphasizes its significant role in community integration. Social integration is strongly associated not only with muscle strength and physical activity levels but also with psychologic well-being and social interactions. Given the multidimensional nature of community integration, interventions targeting social integration are expected to have comprehensive and long-term effects on both physical and mental health.

Given the variety of factors affecting community integration the use of a holistic ICF approach is crucial in identifying and managing the disadvantages that individuals with CHD may experience in terms of community integration.

### Limitations

CHD is a group of heterogeneous diseases. Given this heterogeneity, obtaining an adequate sample size that includes various CHD types and conducting analyses within these groups can help mitigate the impact of heterogeneity when commenting on diagnoses. The strength of our study lies in its ability to evaluate the challenges faced by patients from a multidimensional perspective. By examining individuals with CHD holistically within the ICF framework, we can gain valuable insights into their experiences.

In conclusion, the results of the assessment of individuals with ACHD reveal that community integration is related to the body functions, activity-participation, and environmental-personal factors sub-dimensions of ICF. Conducting multidimensional assessments to determine long-term outcomes that can be improved in this population is becoming increasingly crucial. Identifying interrelated factors through holistic assessments will facilitate the effective management of challenges encountered. Planning comprehensive and individualized interventions that consider all associated factors will significantly contribute to improving ACHD care.

## Supplementary Information

Below is the link to the electronic supplementary material.

Supplementary file1 (DOCX 18 KB)

## Data Availability

Data available on request.
