# Supplementary material for: Investigation of Community Integration in Adults with Congenital Heart Disease Within the Scope of International Classification of Functioning, Disability, and Health
Source: Pediatr Cardiol. 2025 Apr 5;47(2):838–48. doi: 10.1007/s00246-025-03850-4 (PMC12855356; doi:10.1007/s00246-025-03850-4)
Supplement: Supplementary file 1 — Supplementary file1 (DOCX 18 KB) [file 246_2025_3850_MOESM1_ESM.docx]

**Supplemental Table 1. ICF items related to assessment parameters**

|  |  | ICF items | Assessment parameters |
| --- | --- | --- | --- |
| Body Functions | b410 | Heart functions | - 6-minute walk test - Echocardiography |
|  | b455 | Exercise tolerance functions | - 6-minute walk test - Fatigue Severity Scale |
|  | b460 | Sensations associated with cardiovascular and respiratory functions | - 6-minute walk test - Fatigue Severity Scale |
|  | b730 | Muscle power functions | - Muscle Strength |
|  | b780 | Sensations related to muscles and movement functions | - Muscle Strength |
|  | b152 | Emotional functions | - Depression Anxiety Stress Scale |
| Body Structure | s410 | Structure of cardiovascular system | - Echocardiography - Electrocardiography |
| Activity and Participation | d410 | Changing basic body position | - International Physical Activity Questionnaire - Physical Activity Barriers Scale - Multidimensional Quality of Life Scale - Community Integration Questionnaire |
|  | d450 | Walking | - 6-minute walk test - International Physical Activity Questionnaire - Physical Activity Barriers Scale - Community Integration Questionnaire |
|  | d460 | Moving around | - 6-minute walk test - International Physical Activity Questionnaire - Physical Activity Barriers Scale - Community Integration Questionnaire |
|  | d910 | Community life | - International Physical Activity Questionnaire - Physical Activity Barriers Scale - Community Integration Questionnaire |
|  | d920 | Recreation and leisure | - International Physical Activity Questionnaire - Physical Activity Barriers Scale - Community Integration Questionnaire |
| Environmental Factors | e225 | Climate | - Physical Activity Barriers Scale |
|  | e310 | Immediate family | - Physical Activity Barriers Scale - Multidimensional Quality of Life Scale |
|  | e320 | Friends | - Physical Activity Barriers Scale - Multidimensional Quality of Life Scale |
|  | e410 | Individual attitudes of immediate family members | - Physical Activity Barriers Scale - Multidimensional Quality of Life Scale |
|  | e420 | Individual attitudes of friends | - Physical Activity Barriers Scale - Multidimensional Quality of Life Scale |
